# Retinal microvascular alterations in children with amblyopia

**DOI:** 10.1371/journal.pone.0351232

**Published:** 2026-06-08

**Authors:** Christiane Al-Haddad, Hanadi Ibrahim, Nagib Salameh, Zahi Wehbi, Dalia El Hadi, Youssef Zougheib, Ziad Bashshur

**Affiliations:** Department of Ophthalmology, American University of Beirut Medical Center, Beirut, Lebanon; Klinikum Bielefeld gemGmbH, GERMANY

## Abstract

**Purpose:**

To investigate and compare retinal vascular changes, including superficial macular vessel density, foveal avascular zone, and the macular perfusion densities, in amblyopic, fellow, and control eyes using optical coherence tomography angiography.

**Methods:**

In this prospective cross-sectional study, we recruited 78 participants, including 36 with amblyopia and 42 controls, to investigate retinal parameters using optical coherence tomography angiography (OCTA) by comparing amblyopic eyes to both controls and fellow eyes.

**Results:**

A total of 78 patients were enrolled, including 36 with amblyopia (mean age: 9.8 ± 4.6 years) and 42 controls (mean age: 10.0 ± 2.9 years). Among the amblyopic cohort, 18 (50%) had strabismic and 18 (50%) had anisometropic amblyopia. Baseline characteristics, including age, sex distribution, and spherical equivalent, were comparable across groups. Macular OCTA demonstrated higher outer and full macular vessel densities, as well as elevated outer and full regions perfusion densities, in amblyopic eyes relative to control. Optic disc OCTA parameters were comparable between amblyopic eyes and controls. Comparison between amblyopic and fellow eyes showed a significantly smaller macular foveal avascular zone area, with no other significant interocular differences in macular or optic disc OCTA findings. Fellow eyes and control eyes differed only in optic disc perfusion density in the outer region, which was decreased in fellow eyes.

**Conclusion:**

Amblyopic eyes showed increased outer and full macular vessel and perfusion densities compared with control eyes, and a smaller foveal avascular zone area relative to fellow eyes. No significant microvascular differences were found in the optic disc OCTA among groups. These exploratory findings raise the possibility that amblyopia may be associated with subtle macular microvascular alterations, however, confirmatory studies with larger cohorts are needed.

## Introduction

Amblyopia is a neurodevelopmental visual disorder characterized by reduced visual acuity in one or both eyes that cannot be fully corrected with optical lenses. It develops when unbalanced visual input during childhood disrupts normal development of the visual pathway, prompting cortical suppression of input from the affected eye. Common underlying etiologies include strabismus, anisometropia, or visual deprivation due to obstruction or media opacity [1]. Amblyopia remains one of the most prevalent causes of monocular vision loss in children, affecting 1–5% of the population, with a pooled global prevalence of 1.36% reported in 2022 [2,3].

Recent studies have provided new insights into the structural and vascular alterations associated with amblyopia. Magnetic resonance imaging (MRI) studies have shown both structural and functional changes in the visual pathway, particularly the lateral geniculate nucleus and visual cortex [3,4]. At the ocular level, optical coherence tomography (OCT) and optical coherence tomography angiography (OCTA) have become indispensable tools for assessing not only retinal but also choroidal morphology and microvasculature in vivo [5–8]. Several OCT-based studies have reported subtle differences in macular and optic disc parameters between amblyopic and healthy eyes, though results remain inconsistent.[7–11] Some studies observed increased macular thickness and altered retinal nerve fiber layer thickness (RNFLT) in amblyopic eyes, while others found no significant differences [12–21]. More recently, attention has shifted toward the choroid, a vascular layer crucial for outer retinal metabolism. Multiple studies have demonstrated significantly increased choroidal thickness in amblyopic eyes compared with fellow or control eyes, suggesting that amblyopia may involve not only retinal but also deeper vascular alterations [22,23].

Several OCTA studies have investigated retinal microvascular changes in amblyopia; however, findings remain heterogeneous. Multiple reports have shown decreased vessel density in the superficial capillary plexus (SCP) and/or deep capillary plexus (DCP) in amblyopic eyes compared with fellow or control eyes [6,20,24,25], while others found no significant differences in macular vessel density or foveal avascular zone (FAZ) metrics [12,16,21]. Consequently, the current literature lacks consensus on whether amblyopia is associated with reduced, unchanged, or compensatory microvascular changes at the macular and optic disc levels.

Based on prior evidence of increased choroidal thickness and potential compensatory vascular changes in amblyopic eyes from our group and others [7,22,23], we hypothesized that amblyopic eyes would exhibit differences in macular and optic disc perfusion compared with control eyes. The novelty of this study lies in its combined quantitative assessment of both macular and optic disc microvascular parameters using OCTA in a pediatric cohort that includes equal proportions of anisometropic and strabismic amblyopia, with comparisons to both the fellow eyes and age-matched healthy controls.

## Materials and methods

This is a prospective, cross-sectional study that was conducted from 01/11/2021–30/11/2023 at the pediatric ophthalmology clinic of American University of Beirut Medical Center ophthalmology department. Ethical approval from the Institutional Review Board (ID: OPH.CH.06) of the institution was obtained, and all procedures adhered to the Declaration of Helsinki. Written informed consent was obtained before the study from the parents or legal guardians of all participants, and assent forms were collected from children when applicable.

Children aged between 3–18 years with unilateral amblyopia due to anisometropia or strabismus were included. Anisometropia was defined as a cycloplegic spherical equivalent (SE) difference of ≥ 1.0 diopter (D) between the two eyes, and amblyopia was defined as an interocular difference of at least two lines in best-corrected visual acuity (BCVA). Exclusion criteria included deprivational amblyopia of any cause (including ptosis, lid masses, media opacity or structural ocular pathology), any other ocular or systemic condition affecting the retinal or optic nerve vasculature (including glaucoma, retinal disease, and diabetes), previous intraocular surgery or trauma, presence of nystagmus, media opacity interfering with image acquisition, or poor‑quality OCTA scans (signal strength <5/10). The control group consisted of age‑ and sex‑matched healthy children with normal visual acuity (0.0 LogMAR), orthophoria, cycloplegic SE between +2.00 and +4.00 D, and no history of ocular pathology or systemic disease. To avoid inter-eye correlation effects, only the right eye was analyzed for each participant.

All patients underwent a comprehensive eye exam, including BCVA measurement, slit-lamp exam, extraocular motility assessment, intraocular pressure measurement, fundoscopy and cycloplegic retinoscopy after dilation using Cyclopentolate 1% and Tropicamide 1%. Refractive error was reported as SE in diopters, calculated by adding the sum of the sphere power with half the cylinder power. Following pupillary dilation, OCTA images of the macula and optic disc were obtained for each eye using the Cirrus HD-OCT 5000 with AngioPlex OCT module (Carl Zeiss Meditec, Dublin, California, USA; software version 11.0). OCTA parameters were analyzed exclusively in the SCP using the Cirrus HD-OCT built-in automated segmentation algorithm. A 6x6 mm cube scan was obtained for all patients at the macula, and for a subset of participants, an additional scan was performed at the optic disc. The image was divided with the Early Treatment Diabetic Retinopathy Study (ETDRS) grid, defined by three concentric circles of 1, 3, and 6 mm: the central foveal ring, inner macular ring, and outer macular ring [26]. All images were obtained by a single experienced operator and scans showing motion artifacts or low signal strength (<5/10) were immediately repeated until optimal image quality was achieved. Any scan that did not meet image quality criteria was excluded from the final analysis, with only the highest‑quality scans analyzed. Optic disc OCTA analyses were performed on a complete-case basis, including only participants with available high-quality scans. Missing optic disc data were primarily due to inability to obtain acceptable image quality (low signal strength, poor fixation or limited patient cooperation), and such scans were excluded.

Collected data included demographic characteristics including age, sex, type of amblyopia, refractive error, BCVA, and OCTA parameters of the macular and optic disc regions. Amblyopia treatment with patch therapy was collected from the medical records of amblyopic participants. The following parameters were extracted for analysis at the macula: (1) vessel density in the superficial capillary plexus (mm ⁻ ¹; total length of perfused vasculature per unit area) across the central, inner, outer, and full macular regions; (2) perfusion density (%; percentage of perfused area per unit area) in the same macular subfields; (3) foveal avascular zone (FAZ) metrics, including area (mm²), perimeter (mm), and circularity (unitless index, ranging from 0 to 1, where 1 indicates a perfect circle). FAZ parameters were analyzed only when automatically detected by the OCTA device. For optic disc imaging, only vessel and perfusion densities were assessed within the whole disc, peripapillary, inner, and outer regions. Comparisons were performed between (i) amblyopic and fellow eyes, (ii) amblyopic and control eyes, (iii) fellow eyes of amblyopic patients and control eyes, and (iv) strabismic and anisometropic amblyopic eyes.

### Statistical analysis

Data analysis was conducted using IBM SPSS Statistics for Windows, version 28.0 (IBM Corp., NY, USA). Categorical variables were summarized as frequencies and percentages, and compared using the chi-square test or Fischer's exact test as appropriate. Normality of continuous variables was evaluated using the Shapiro-Wilk test. Normally distributed data were presented as mean ± standard deviation (SD) and compared using independent t-test. A p‑value < 0.05 was considered statistically significant. Power analysis using G*Power from a prior study [14] indicated a sample size of 35 participants per group (α = 0.05; power = 95%). A post‑hoc power analysis based on observed differences in outer macular vessel density between amblyopic and control eyes, demonstrated a power of 0.81 at α = 0.05. Given that multiple OCTA parameters were derived from the same eye within each scan, these measures may therefore be correlated. Accordingly, subgroup and regional p-values should be interpreted with caution.

## Results

### Demographics

A total of 78 patients were recruited, including 36 (46.2%) with amblyopia and 42 (53.8%) controls. The mean age of the amblyopic group was 9.8 ± 4.6 years, and 10 ± 2.9 years in the control group, with a male-to-female ratio of 1.8:1 in the amblyopia group and 1.9:1 in the control group. There were no significant differences in age, gender distribution, or SE between groups, whereas BCVA was significantly worse in amblyopic eyes. The mean BCVA was 0.34 ± 0.18 in amblyopic eyes compared to 0.0 ± 0.0 in controls, with mean SE of +2.51 ± 3.86D and +1.77 ± 1.15D, respectively. Among amblyopic patients, 18 (50%) had strabismic amblyopia and 18 (50%) had anisometropic amblyopia. All had a history of occlusion therapy; 26 (72%) were receiving active patching at the time of assessment, with a mean patching duration of 13 ± 10 months. Macular OCTA was performed in all participants, whereas optic disc OCTA was obtained in 28 amblyopic patients (78%) and 31 controls (74%). The mean signal strength did not differ significantly among groups for either macular or optic disc OCTA scans. Patient demographics and clinical characteristics are presented in Table 1.

**Table 1:** Patient demographics and clinical characteristics.

Characteristics	Amblyopic eye (n = 36)	Fellow eye (n = 36)	Control eye (n = 42)
Age (years), mean ± SD	9.8 ± 4.6	9.8 ± 4.6	10 ± 2.9
Gender (M: F)	1.8: 1	1.8: 1	1.9: 1
Performed optic disc OCTA, n (%)	28 (78%)	28 (78%)	31 (74%)
Amblyopia type, n (%):			
Strabismic	18 (50%)	–	–
Anisometropic	18 (50%)	–	–
BCVA (LogMAR)	0.34 ± 0.18	0.06 ± 0.10	0.0 ± 0.0
Spherical equivalent (D)	+2.51 ± 3.86	+2.10 ± 2.52	+1.77 ± 1.15
Signal strength index (0–10)			
Macular OCTA	7.4 ± 1.9	7.9 ± 1.7	7.3 ± 1.4
Optic disc OCTA	7.8 ± 1.4	7.8 ± 1.6	7.2 ± 1.6

*BCVA: best-corrected visual acuity; D: diopters; OCTA: optical coherence tomography angiography; SD: standard deviation; Signal strength: image quality score (range 0–10), with higher values indicating better image quality.*

### Amblyopic eye vs control

On macular OCTA, amblyopic eyes showed significantly increased vessel density in the outer (p = 0.02) and full macular regions (p = 0.03) compared to control eyes. Vessel density in the inner macular region showed a non-significant trend toward higher values in amblyopic eyes (p = 0.06). Regarding FAZ metrics (area, perimeter, and circularity), no significant differences were detected. In terms of macular perfusion density, amblyopic eyes showed higher outer (p = 0.02) and full (p = 0.03) perfusion densities, while central and inner regions did not differ significantly.

Regarding optic disc OCTA parameters, no significant differences were observed in vessel or perfusion density between amblyopic and control eyes. OCTA results comparing amblyopic and control eyes are detailed in Table 2.

**Table 2:** Comparison of optical coherence tomography angiography macula and nerve parameters between amblyopic and control eyes.

	Amblyopic eye	Control eye	p-value
**OCTA Macula (n)**	36	42	
**Vessel Density, mean ± SD**			
Central (mm^-1^)	6.44 ± 4.20	5.58 ± 3.93	0.36
Inner (mm^-1^)	15.19 ± 2.77	13.68 ± 3.91	0.06
Outer (mm^-1^)	16.44 ± 2.62	14.75 ± 3.54	**0.02**
Full (mm^-1^)	15.87 ± 2.58	14.41 ± 3.01	**0.03**
Foveal Avascular Zone*, mean ± SD			
Area (mm^2^)	0.17 ± 0.12	0.19 ± 0.20	0.66
Perimeter (mm)	1.73 ± 0.58	1.73 ± 0.86	0.97
Circularity	0.67 ± 0.11	0.69 ± 0.09	0.35
**Perfusion Density, mean ± SD**			
Central (%)	15 ± 9	37 ± 159	0.40
Inner (%)	35 ± 9	32 ± 10	0.23
Outer (%)	40 ± 6	37 ± 7	**0.02**
Full (%)	39 ± 6	35 ± 8	**0.03**
**Optic disc OCTA (n)**	28	31	
**Vessel Density, mean ± SD**			
Central (mm^-1^)	9.17 ± 4.66	10.31 ± 5.12	0.28
Inner (mm^-1^)	18.09 ± 2.58	18.26 ± 1.32	0.30
Outer (mm^-1^)	17.66 ± 2.79	17.34 ± 1.57	0.68
Full (mm^-1^)	17.51 ± 2.60	17.38 ± 1.17	0.51
**Perfusion Density, mean ± SD**			
Central (%)	24 ± 13	27 ± 14	0.46
Inner (%)	47 ± 7	46 ± 7	0.52
Outer (%)	44 ± 7	42 ± 6	0.30
Full (%)	44 ± 7	43 ± 6	0.36

OCTA: optical coherence tomography angiography; FAZ: foveal avascular zone; SD: standard deviation.

* FAZ metrics were computed only for scans with successful automatic detection (n = 26 amblyopic; n = 30 control).

Subgroup comparison between strabismic and anisometropic amblyopic eyes showed no statistically significant differences in any macular or optic disc OCTA parameter, including vessel density, perfusion density, or FAZ metrics (all p > 0.05).

### Amblyopic vs fellow eye

When evaluating macular vessel density using OCTA, no statistically significant differences were found between the amblyopic and fellow eyes across the central, inner, outer, and full regions. Likewise, macular perfusion density was comparable between eyes across all regions. Analysis of FAZ metrics revealed a smaller FAZ area in amblyopic eyes (p = 0.02), while perimeter and circularity did not differ significantly between eyes.

For optic disc OCTA measurements, no statistically significant differences were observed between amblyopic and fellow eyes in vessel or perfusion density across all evaluated regions. OCTA parameter comparisons between amblyopic and fellow eyes are provided in Table 3.

**Table 3:** Comparison of optical coherence tomography angiography macula and nerve parameters between amblyopic and fellow eyes.

	Amblyopic eye	Fellow eye	p-value
**OCTA Macula (n)**	36	36	–
**Vessel Density, mean ± SD**			
Central (mm^-1^)	6.44 ± 4.20	6.98 ± 4.22	0.47
Inner (mm^-1^)	15.19 ± 2.77	14.88 ± 4.05	0.66
Outer (mm^-1^)	16.44 ± 2.62	15.99 ± 2.83	0.32
Full (mm^-1^)	15.87 ± 2.58	15.49 ± 2.99	0.45
Foveal Avascular Zone*, mean ± SD			
Area (mm^2^)	0.17 ± 0.12	0.21 ± 0.13	**0.02**
Perimeter (mm)	1.73 ± 0.58	1.83 ± 0.56	0.17
Circularity	0.67 ± 0.11	0.72 ± 0.09	0.08
**Perfusion Density, mean ± SD**			
Central (%)	15 ± 9	15 ± 9	0.68
Inner (%)	35 ± 9	38 ± 020	0.33
Outer (%)	40 ± 6	39 ± 8	0.51
Full (%)	39 ± 6	38 ± 8	0.61
**Optic disc OCTA (n)**	28	28	–
**Vessel Density, mean ± SD**			
Central (mm^-1^)	9.17 ± 4.66	10.24 ± 5.64	0.17
Inner (mm^-1^)	18.09 ± 2.58	18.33 ± 1.21	0.65
Outer (mm^-1^)	17.66 ± 2.79	17.97 ± 1.71	0.64
Full (mm^-1^)	17.51 ± 2.60	17.82 ± 1.48	0.61
**Perfusion Density, mean ± SD**			
Central (%)	24 ± 13	27 ± 15	0.11
Inner (%)	47 ± 7	47 ± 3	0.74
Outer (%)	44 ± 7	45 ± 4	0.58
Full (%)	44 ± 7	45 ± 4	0.55

OCTA: optical coherence tomography angiography; FAZ: foveal avascular zone; SD: standard deviation.

* FAZ metrics were computed only for scans with successful automatic detection (n = 26 amblyopic; n = 27 fellow).

### Fellow eye vs control

In the comparison between fellow eyes of amblyopic patients and control eyes, there were no statistically significant differences in macular vessel density and perfusion density across the central, inner, outer, or full macular regions. The FAZ parameters were also comparable between groups.

Regarding optic disc measurements, fellow eyes mostly showed similar vessel and perfusion density parameters compared to control eyes, with only an isolated higher outer perfusion density compared to controls (45 ± 4% vs 42 ± 6%, p = 0.048); Comparisons of macular and optic nerve OCTA parameters between fellow and control eyes are detailed in Table 4.

**Table 4:** Comparison of optical coherence tomography angiography macula and nerve parameters between fellow and control eyes.

	Fellow eye	Control eye	p-value
**OCTA Macula (n)**	36	42	
**Vessel Density, mean ± SD**			
Central (mm^-1^)	6.98 ± 4.22	5.58 ± 3.93	0.14
Inner (mm^-1^)	14.88 ± 4.05	13.68 ± 3.91	0.19
Outer (mm^-1^)	15.99 ± 2.83	14.75 ± 3.54	0.10
Full (mm^-1^)	15.49 ± 2.99	14.41 ± 3.01	0.12
Foveal Avascular Zone*, mean ± SD			
Area (mm^2^)	0.21 ± 0.13	0.19 ± 0.20	0.67
Perimeter (mm)	1.83 ± 0.56	1.73 ± 0.86	0.62
Circularity	0.72 ± 0.09	0.69 ± 0.09	0.39
**Perfusion Density, mean ± SD**			
Central (%)	15 ± 9	37 ± 159	0.42
Inner (%)	38 ± 20	32 ± 10	0.10
Outer (%)	39 ± 8	37 ± 7	0.09
Full (%)	38 ± 8	35 ± 8	0.10
**Optic disc OCTA (n)**	28	31	
**Vessel Density, mean ± SD**			
Central (mm^-1^)	10.24 ± 5.64	10.31 ± 5.12	0.96
Inner (mm^-1^)	18.33 ± 1.21	18.26 ± 1.32	0.82
Outer (mm^-1^)	17.97 ± 1.71	17.34 ± 1.57	0.14
Full (mm^-1^)	17.83 ± 1.48	17.38 ± 1.17	0.20
**Perfusion Density, mean ± SD**			
Central (%)	27 ± 15	27 ± 14	0.85
Inner (%)	47 ± 3	46 ± 7	0.24
Outer (%)	45 ± 4	42 ± 6	**0.048**
Full (%)	45 ± 4	43 ± 6	0.054

*OCTA: optical coherence tomography angiography; FAZ: foveal avascular zone; SD: standard deviation.*

* FAZ metrics were computed only for scans with successful automatic detection (n = 27 fellow; n = 30 control).

Boxplot distributions of the OCTA parameters are shown in Supplementary S1–S5 Figs.

## Discussion

This study demonstrated the presence of microvascular alterations in the macular region of amblyopic eyes compared to controls. Amblyopic eyes exhibited significantly higher vessel and perfusion densities in the outer and full macular regions, while FAZ parameters were comparable between groups. When amblyopic eyes were compared with their fellow eyes, macular vascular densities did not differ significantly, although amblyopic eyes showed a smaller FAZ area. No significant differences were observed in optic disc vessel or perfusion densities across comparisons. Fellow eyes demonstrated significantly higher optic disc outer perfusion density compared to controls, with otherwise similar remaining characteristics.

Multiple studies have investigated the retinal microvascular changes in amblyopia using OCTA [7,8]. Several studies, in contrast to our findings, reported decreased capillary vessel density (CVD) in superficial capillary plexus (SCP) and deep capillary plexus (DCP) in amblyopic eyes of children compared to healthy controls [6], but no significant difference in FAZ measurements [24,25]. Others demonstrated significantly lower CVD in both SCP and DCP in amblyopic eyes compared to fellow eyes and healthy controls, along with wider FAZ measurements in amblyopic and fellow eyes [20]. A meta-analysis of 17 studies confirmed an overall reduction in vessel density in amblyopic eyes but highlighted substantial variability depending on scan size, plexus analyzed, and control selection; differences were more consistently detected in 6 × 6-mm scans, and findings were less consistent when fellow eyes were used as controls.[27] These data indicate that age, amblyopia subtype, scan protocol, plexus selection, and magnification correction significantly influence OCTA metrics. In our study, analysis was restricted to the SCP only (Cirrus HD-OCT 5000 AngioPlex, 6 × 6-mm ETDRS grid, automated segmentation, no axial-length correction) in a balanced pediatric cohort (50% strabismic/50% anisometropic) with moderate amblyopia. These methodological differences, particularly SCP-only analysis on a Zeiss platform (versus predominantly Optovue SCP + DCP in prior studies) and balanced subtypes, likely explain the increased outer and full macular vessel and perfusion densities we observed. These studies suggest that reduced microvascular density may be related to the decreased size and number of retinal ganglion cells. However, the exact relationship between vascular changes and amblyopia remains unclear [6,18,20,24].

Some studies have reported a difference in foveal vessel densities between the amblyopic and fellow eyes in children and adults [12,16]. In contrast, and similar to our findings, a systemic review found no significant difference regarding the FAZ and foveal thickness between the amblyopic and fellow eyes in children [21]. However, whereas that review and several previous reports described a reduction in vessel density, our study demonstrated increased vessel and perfusion densities in the outer and full macular regions of amblyopic eyes compared with control eyes. In addition, we found that the FAZ area was significantly smaller in amblyopic eyes than in their fellow eyes, while the other FAZ parameters were comparable. This may reflect delayed foveal maturation or incomplete macular development rather than true microvascular loss, consistent with prior OCT evidence of immature macular features in amblyopia.[7] Nevertheless, these findings collectively suggest that the fellow eye in children with amblyopia may not be equivalent to a healthy eye in children with no amblyopia. Additionally, Ye et at. demonstrated high interocular symmetry in the FAZ area and perimeter in normal healthy subjects [28]. Therefore, the fellow eye of individuals with amblyopia may not serve as an appropriate control when studying vascular alterations, as amblyopia may be associated with subtle bilateral developmental changes despite its unilateral clinical presentation.

In contrast to other literature, our study revealed increased vascular density in amblyopic eyes compared to control eyes. This may be related to the increased choroidal thickness in amblyopia [22]. In the earlier study using HD-OCT, our group demonstrated a significant increase in choroidal thickness in amblyopic eyes compared to controls. Considering the close anatomical relationship between the choroid and retinal vasculature, it is reasonable to hypothesize that a thicker choroid in amblyopic eyes would be associated with an increased blood supply. This could potentially contribute to increased vascular density as observed in our OCTA macular measurements. Our earlier work using OCT and segmenting the retinal layers showed changes in the macular ultrastructure commensurate with a less mature and thicker macula, in turn corresponding to a higher vascular supply [7,22,23].

In our current study, we found no statistically significant difference in the microvasculature of the optic disc between amblyopic and control eyes, nor between amblyopic and fellow eyes. However, fellow eyes demonstrated a mild significant increase in ONH outer perfusion density compared with controls (p = 0.048), while all other ONH parameters remained comparable. This isolated finding has no clear clinical relevance. It may reflect a type I error given the marginal p-value and modest effect size, or subtle compensatory changes in the non-amblyopic eye. Importantly, it still supports the evidence that fellow eyes in children with unilateral amblyopia are not fully equivalent to healthy control eyes [11,28]. This observation aligns with recent 3D-OCTA studies demonstrating subclinical bilateral microvascular alterations in fellow eyes, including reduced vessel volume and skeleton density as well as increased vessel diameter and tortuosity, compared with true healthy controls; and with multiple reports emphasizing that fellow eyes should not be considered normal reference eyes [11,29–31]. This is particularly important given that healthy pediatric eyes generally show good interocular symmetry for FAZ and vessel density [32]. Consequently, using the fellow eye as a “normal” control in OCT or OCTA studies of amblyopia should be approached with caution, as it may mask subtle bilateral microvascular changes.

Although we found no statistically significant differences in amblyopic eyes, previous studies have reported a marginally significant decrease in the optic disc flow area and vessel density was reported, with higher RNFL thickness in amblyopic eyes [9]. Similar patterns were noted in adult amblyopia cohorts using OCTA, where small vessels and overall capillary density inside the optic disc were reduced in amblyopic eyes relative to both fellow and control eyes [10]. In another study, the capillary vessel density inside the optic disc was decreased in amblyopic eyes compared to fellow and control eyes, whilst no statistical significance when comparing this vessel density in the whole image, the peripapillary region, and the superior and inferior hemisphere [20]. A recent study by Simsek et al. (2025) evaluating pediatric patients with anisometropic, strabismic, and deprivation amblyopia reported significantly lower whole-image, foveal, and parafoveal vessel densities in both SCP and DCP compared to controls. Subgroup analysis in our study showed no significant differences in any macular or optic disc OCTA parameters between anisometropic and strabismic amblyopia, consistent with prior reports [33]. The differences between these findings and our results may be explained by differences in amblyopia subtype distribution and inclusion of both SCP and DCP. Clinically, these findings indicate that OCTA vascular parameters in amblyopia vary by subtype and retinal layer, limiting their reliability as standalone biomarkers and warranting cautious interpretation in clinical practice.

One limitation of the study is the potential impact of poor fixation in amblyopic eyes on OCTA imaging. Due to the impaired fixation commonly observed in individuals with amblyopia, the acquisition of accurate images can be challenging, particularly in cases of deep amblyopia. As a result, there may be instances where the OCTA technology failed to capture the complete extent of vascular density in amblyopic eyes. To minimize this limitation, scans showing motion artifacts or low signal strength were immediately repeated until optimal image quality was achieved, otherwise the patient was excluded. Additionally, FAZ characteristics should be interpreted with caution, as they were not consistently calculated by the OCTA device in all scans, which may have introduced measurement variability and reduced the precision of FAZ-related comparisons. Since the study focused exclusively on the superficial capillary plexus using Cirrus AngioPlex, the deep capillary plexus was not evaluated; this limits direct comparison with studies analyzing both plexuses but is consistent with the device’s standard segmentation protocol. A further limitation is the absence of axial length measurements that can influence ocular magnification for OCTA metrics; however, spherical equivalent was comparable across groups, which reduced the potential for systematic bias. Finally, most amblyopic participants (72%) were still undergoing occlusion therapy at the time of assessment, a factor that may have influenced microvascular measurements, as suggested by previous studies.[34,35]. Although the study achieved sufficient statistical power (81%) and an adequate sample size, the findings should still be interpreted with caution, as variability between studies may reflect methodological differences or heterogeneous patient populations. Prospective longitudinal studies are still needed to investigate the changes in the macular and optic disc microvasculature of amblyopic children before and after treatment to determine a possible causative relationship. An additional statistical consideration is that multiple OCTA measurements per eye were analyzed separately, which may have introduced intra-eye correlation and inflated statistical significance. Therefore, these regional findings should be interpreted with caution and considered exploratory.

In conclusion, this study provided exploratory evidence suggesting that amblyopic eyes may exhibit increased macular vessel and perfusion densities, particularly in the outer and full regions, together with a smaller FAZ area compared with control eyes, while optic nerve head parameters remain largely unaffected. These findings raise the possibility that amblyopia may be associated with localized alterations in macular microvasculature rather than generalized ocular circulatory changes, providing preliminary insight into the structural and functional mechanisms underlying the condition. Nonetheless, they should be interpreted with caution given the exploratory statistical approach and the potential for intra-eye correlation to influence p-values. Confirmatory studies employing larger cohorts are needed to validate these trends and clarify the clinical relevance of microvascular changes in amblyopia.

## Supporting information

S1 Fig Boxplot of full macular vessel density among amblyopic, fellow, and control eyes. (PNG)

S2 Fig Boxplot of foveal avascular zone area among amblyopic, fellow, and control eyes. (PNG)

S3 Fig Boxplot of full perfusion vessel density among amblyopic, fellow, and control eyes. (PNG)

S4 Fig Boxplot of optic disc vessel density among amblyopic, fellow, and control eyes. (PNG)

S5 Fig Boxplot of optic disc perfusion density among amblyopic, fellow, and control eyes. (PNG)

S6 Data Dataset. Anonymized dataset. (XLSX)
