# Supplementary figures and images for: Retinal microvascular alterations in children with amblyopia
Source: PLoS One. 2026 Jun 8;21(6):e0351232. doi: 10.1371/journal.pone.0351232 (PMC13245761; doi:10.1371/journal.pone.0351232)

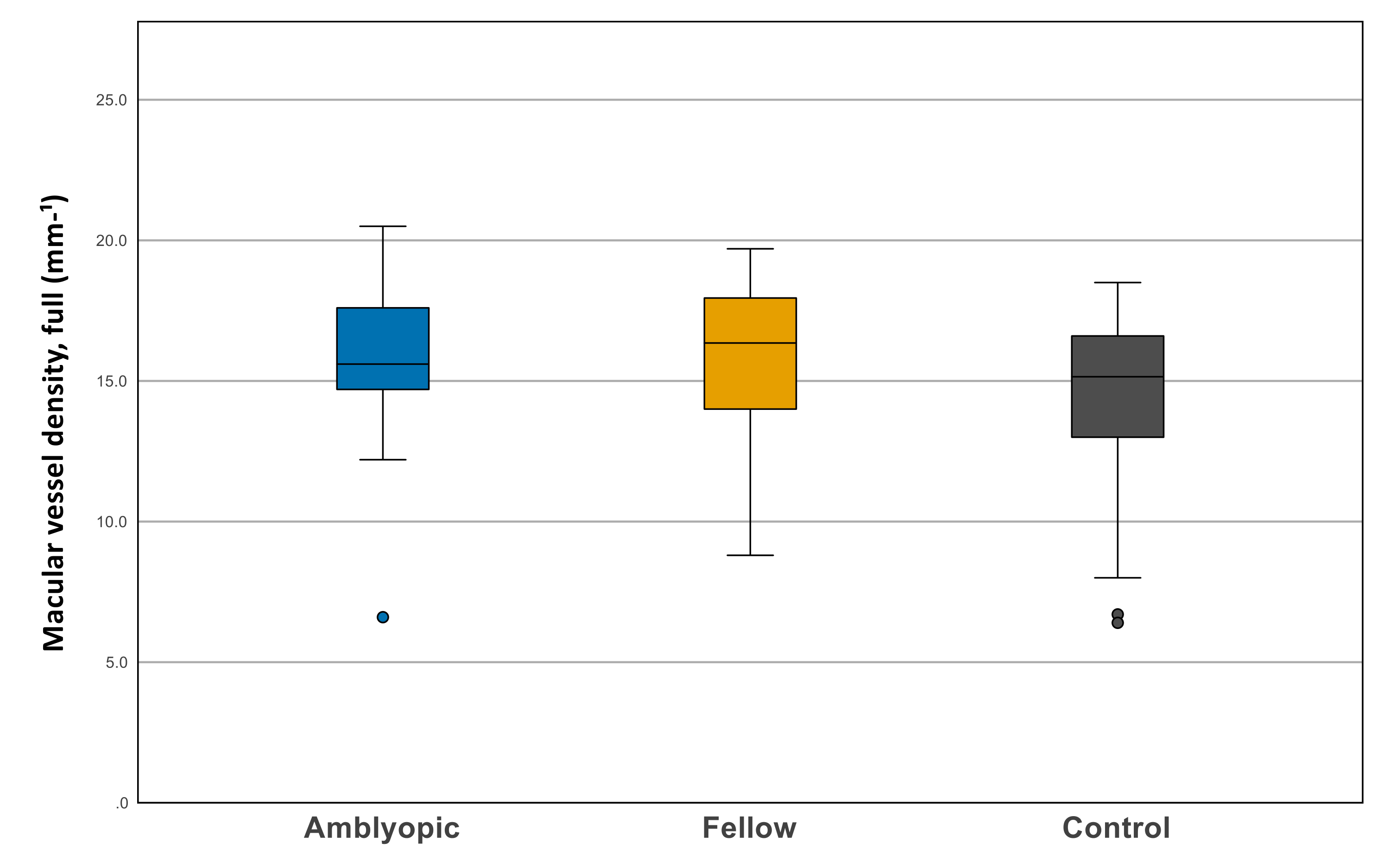

Supplement: S1 Fig — (PNG) [file pone.0351232.s001.png]

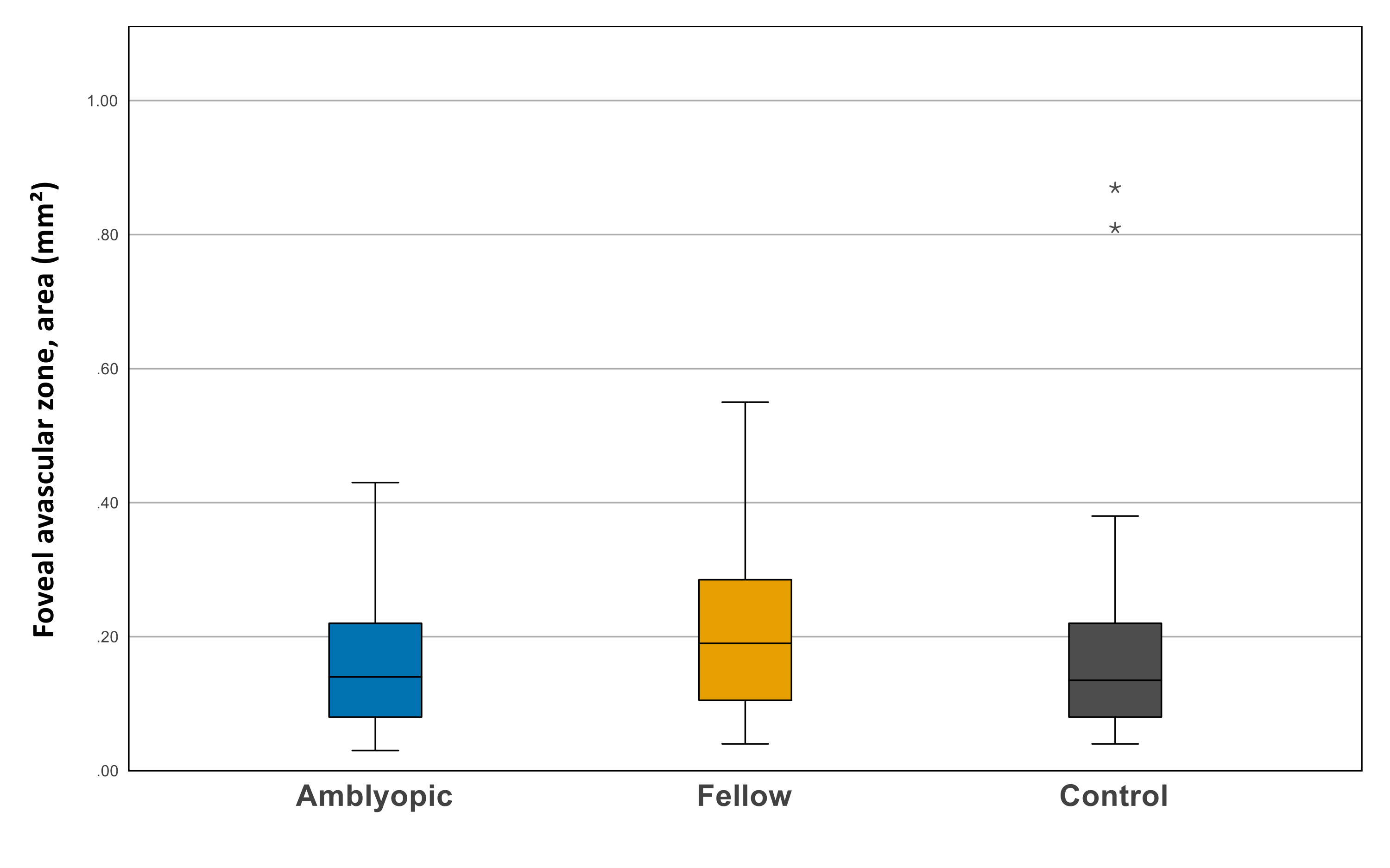

Supplement: S2 Fig — (PNG) [file pone.0351232.s002.png]

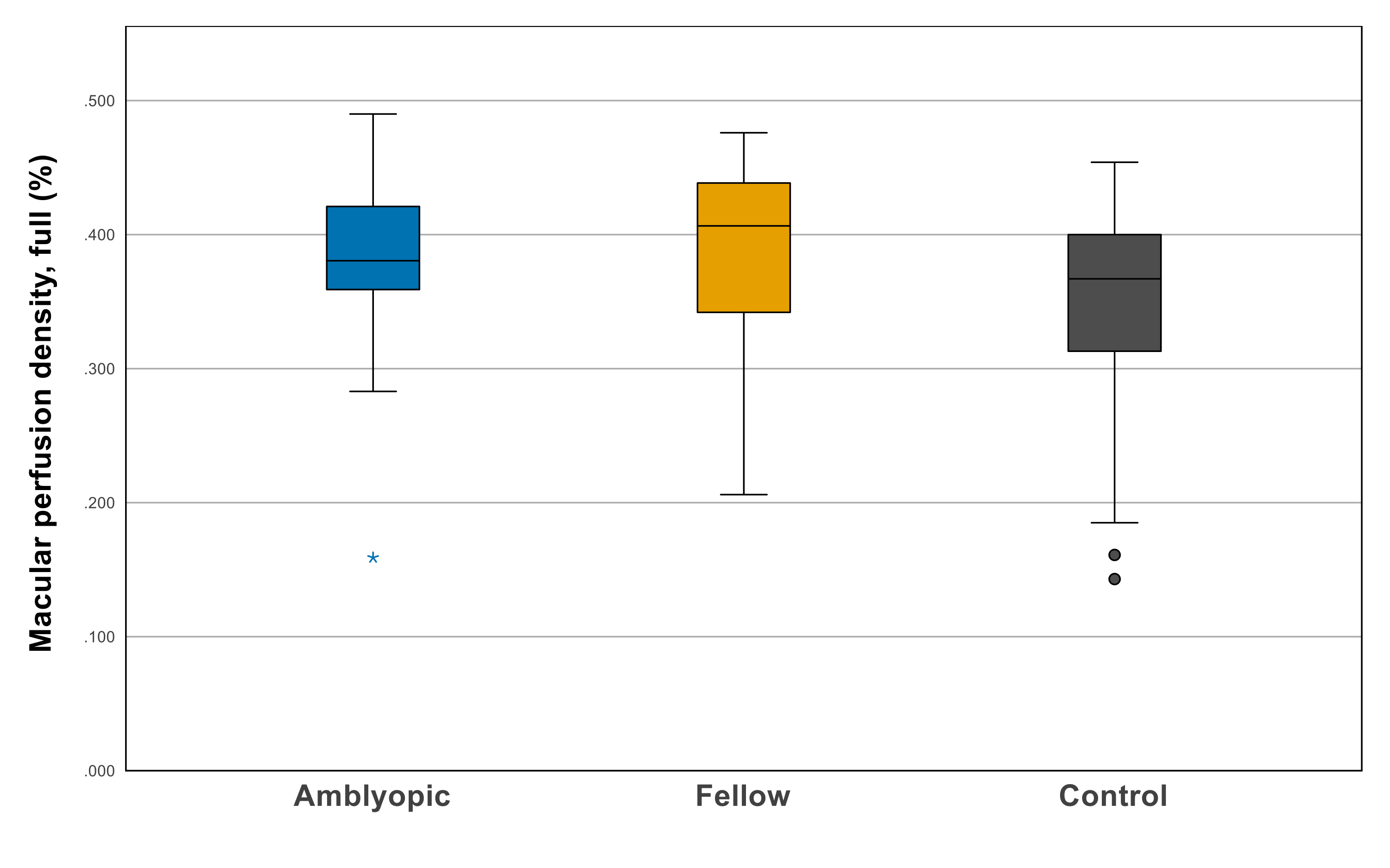

Supplement: S3 Fig — (PNG) [file pone.0351232.s003.png]

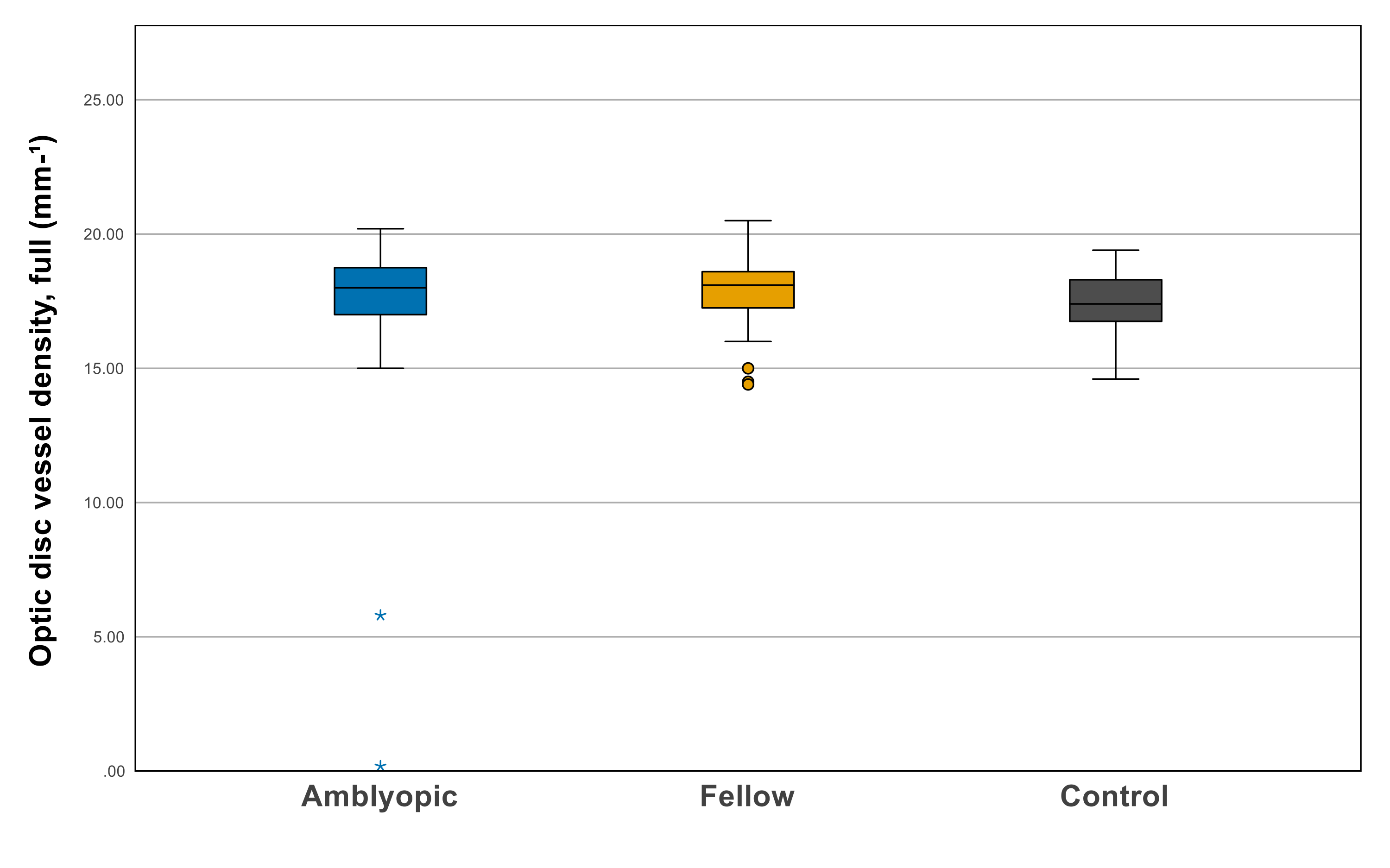

Supplement: S4 Fig — (PNG) [file pone.0351232.s004.png]

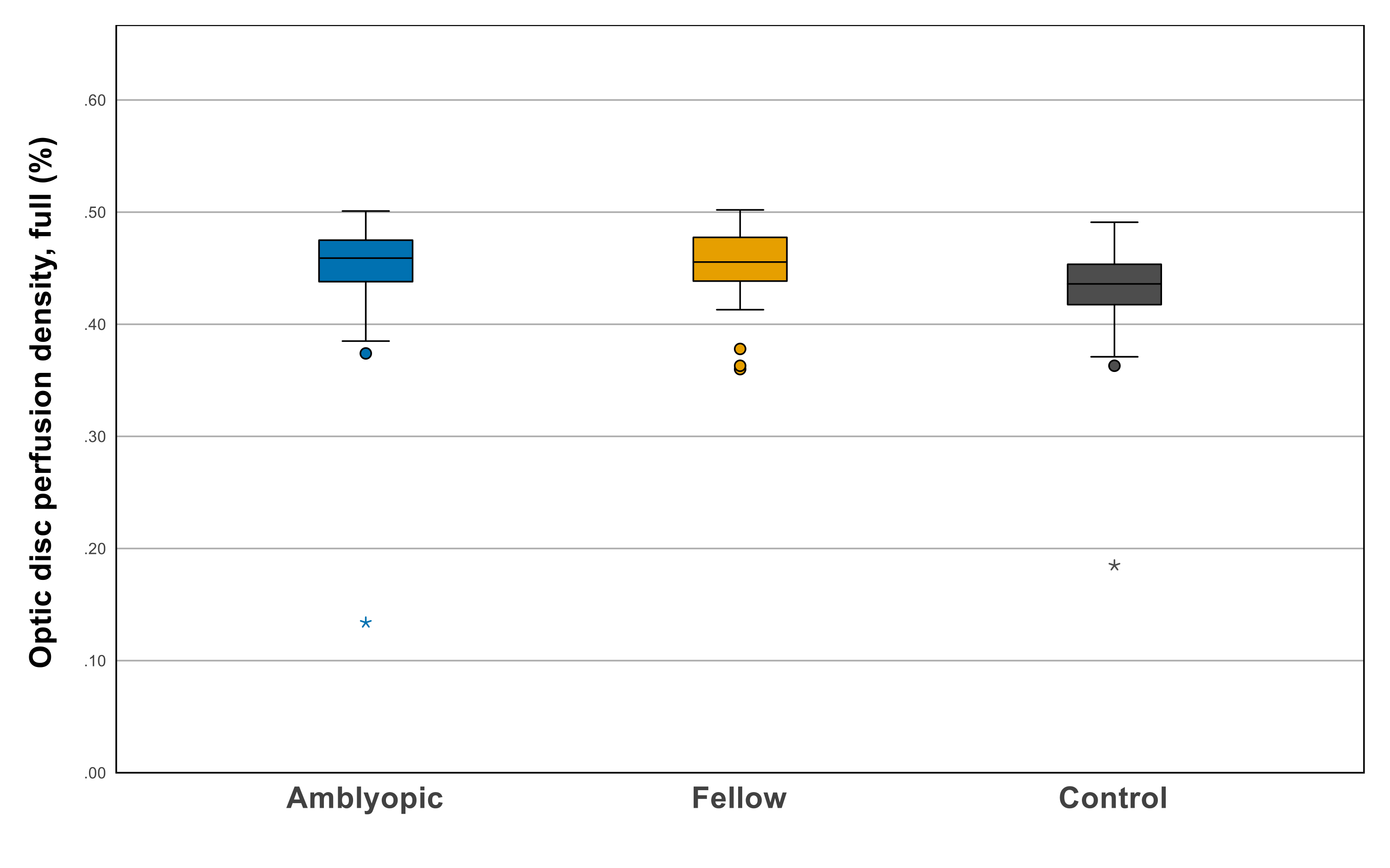

Supplement: S5 Fig — (PNG) [file pone.0351232.s005.png]
